# H_2_S, Polysulfides, and Enzymes: Physiological and Pathological Aspects

**DOI:** 10.3390/biom10040640

**Published:** 2020-04-21

**Authors:** Noriyuki Nagahara, Maria Wróbel

**Affiliations:** 1 Nippon Medical School, Isotope Research Institute, 1-1-5 Sendagi, Bunkyo-ku, Tokyo 113-8602, Japan; 2 Faculty of Medicine, Jagiellonian University Medical College, Kopernika 7 Cracow, 31-034 Krakow, Poland

**Keywords:** cystathionine β-synthase, cystathionine γ-lyase, thiosulfate sulfurtransferase, H_2_S, 3-mercaptopyruvate sulfurtransferase, polysulfides

## Abstract

We have been studying the general aspects of the functions of H_2_S and polysulfides, and the enzymes involved in their biosynthesis, for more than 20 years. Our aim has been to elucidate novel physiological and pathological functions of H_2_S and polysulfides, and unravel the regulation of the enzymes involved in their biosynthesis, including cystathionine β-synthase (EC 4.2.1.22), cystathionine γ-lyase (EC 4.4.1.1), thiosulfate sulfurtransferase (rhodanese, EC 2.8.1.1), and 3-mercaptopyruvate sulfurtransferase (EC 2.8.1.2). Physiological and pathological functions, alternative biosynthetic processes, and additional functions of H_2_S and polysulfides have been reported. Further, the structure and reaction mechanisms of related enzymes have also been reported. We expect this issue to advance scientific knowledge regarding the detailed functions of H_2_S and polysulfides as well as the general properties and regulation of the enzymes involved in their metabolism. We would like to cover four topics: the physiological and pathological functions of H_2_S and polysulfides, the mechanisms of the biosynthesis of H_2_S and polysulfides, the properties of the biosynthetic enzymes, and the regulation of enzymatic activity. The knockout mouse technique is a useful tool to determine new physiological functions, especially those of H_2_S and polysulfides. In the future, we shall take a closer look at symptoms in the human congenital deficiency of each enzyme. Further studies on the regulation of enzymatic activity by in vivo substances may be the key to finding new functions of H_2_S and polysulfides.

## 1. Enzyme Production of H_2_S and Polysulfides

Cystathionine β-synthase (CBS) was first reported to produce H_2_S and polysulfides by Abe and Kimura in 1996 [1]. Further, cystathionine γ-lyase (CGL) was reported by Hosoki et al. [2], and 3-mercaptopyruvate sulfurtransferase (MST) was reported by Shibuya et al. [3,4,5], Mikami et al. [6,7], Modin et al. [8], Yadav et al. [9], Kimura et al. [10], and Nagahara et al. [11]. Thiosulfate sulfurtransferase (TST) was reported by Mikami et al. [7] and Kimura et al. [10]. These enzymes catalyze a transsulfuration reaction from a sulfur-donor substrate to a sulfur acceptor substrate. Then, the persulfurated or polysulfurated substrate is reduced to produce H_2_S and polysulfides during this reaction.

On the other hand, Nagahara et al. [11] recently demonstrated in vitro that MST transfers a sulfur atom from 3-mercptopyruvate (MP) to the catalytic site cysteine to form stable persulfide (polysulfide) as a reaction intermediate. It is interesting that as an alternative production process, thiol-containing compounds attack the persulfide (polysulfide) formed at the catalytic site and a new persulfide (polysulfide) molecule is formed at the thiol-containing compound. Then, dithiol is reduced by thioredoxin (Trx) or dihydrolipoic acid to release H_2_S or polysufides. This process may be autoreduction. Yadav et al. [10] performed enzyme kinetics analysis for human MST in the production process of hydrogen disulfide.

## 2. Physiological Functions of H_2_S and Polysulfides

Kimura reviewed the physiological activities of H_2_S and polysulfides in 2016 [12]. The functions of H_2_S and polysulfides are summarized in Table 1 and Table 2, respectively, [1,2,13,14,15,16,17,18,19,20,21,22,23,24,25,26,27,28,29,30,31,32].

Sulfane-sulfur binding proteins (SSBPs), ubiquitous in cells and tissues, can be regarded as potentially H_2_S-releasing molecules when under proper redox conditions and affected by specific stimuli that induce their release. Three sulfurtransferases, i.e., CGL, MST, and TST, carry sulfane sulfur, which can be released as H_2_S/HS^−^ [33]. The released H_2_S can be enzymatically oxidized to sulfane sulfur by sulfide quinone oxidoreductase with an acceptor of sulfane sulfur, such as GSH [34].

To understand the physiological function of sulfane sulfur, its levels in biological samples (tissues, cell cultures) were determined using the reaction with cyanide, and subsequently investigating the thiocyanate yield of the complex with Fe^3+^, which is detectable by spectrophotometry [35]. Although the method is not very sensitive, it showed that sulfane sulfur levels were quite similar in various animal tissues [36,37] and in murine macrophages despite stimulation with lipopolysaccharide and interferon-γ [38]. Thus, an argument may be put forward about homeostasis of sulfane sulfur levels in biological systems. Moreover, a negative feedback regulation between CBS and CTH was suggested by Nandi and Mishra [39] and confirmed by Bronowicka et al. [38]. The adaptive cellular response to stimulation with both IFNγ and LPS caused a decreased level of H_2_S-associated low CBS expression and increased CTH expression.

The adaptive cellular response to electrophiles (electron-deficient species) represented by heavy metal ions [40] involves sulfane sulfur atoms of numerous SSBPs. Protection against electrophilic stresses involves persulfides rather than thiol groups because of their higher nucleophilicity [41]. Electrophiles are captured by reactive persulfide/polysulfide species, resulting in formation of their sulfur adducts [42]. In bovine aortic endothelial cells, CSE knockdown potentiated Cd-induced cytotoxicity but CSE overexpression provided protection [43]. In vivo experiments showed that CSE-knockout mice were sensitive to Cd-induced hepatotoxicity [44]. Adaptive changes in the activity and expression of CGL, MST, and TST in various frog tissues in response to exposure to lead, mercury, and cadmium confirmed the protective function of these enzymatic proteins against electrophilic stress [45,46].

## 3. Possible Production of Other Sulfur-Containing Substances, Sulfur Oxides

Physiological roles of sulfur dioxide were reported by Liu et al. [47] in 2016 and include vasorelaxation [48,49,50] as well as myocardial injury [48]. MALDI-TOF-MS analysis provided supporting evidence for sulfur oxide production (SO, SO_2_, and SO_3_) in the redox cycle of sulfane sulfur as a reaction intermediate of MST by Nagahara et al. [51] in 2012. The persulfurated catalytic site cysteine was oxidized to form Cys-thiosulfenate (Cys-Sγ-SO), Cys-thiosulfinate (Cys-Sγ-SO_2_), and Cys-thiosulfonate (Cys-Sγ-SO_3_). Reducing agents such as DTT and Trx produce sulfur oxides [51].

## 4. Knockout of H_2_S and Polysulfides-Producing Enzymes

Knockout (KO) technique is a good tool to clarify the physiological functions of proteins. Congenial deficiency of CBS causes hyperhomocysteinemia or homocystinuria in humans. Watanabe et al. produced CBS-KO mice [52], and the mice were afflicted with chronic renal dysfunction. The mice showed growth retardation and died within 5 weeks.

Congenial deficiency of CGL causes cystathioninuria in humans. CGL-KO mice were produced by Yang et al. [53], and the mice displayed low levels of H_2_S associated with hypertension.

Congenial deficiency of MST causes mercaptolactate-cysteine disulfidria in humans. MST-KO mice were produced by Nagahara et al. [54]; however, mercaptolactate-cysteine disulfiduria has not been examined. Peleli et al. [55] recently reported that MST-KO mice were protected against ischemic reperfusion of the heart. Nasi et al. [56] reported that mice showed accelerated joint calcification and osteoarthritis due to an increase in chondrocyte mineralization. Interestingly, these two findings indicate that MST demonstrates both good and bad effects on living organisms.

TST-KO mice have not been obtained; however, a double KO mouse for both TST and MST has been produced (unpublished data).

## 5. Regulation of Enzymatic Activity by In Vivo Substances

These four enzymes have been reported to regulate enzymatic activities in vivo. CBS activity is inhibited by CO [57], NO [58], L-cystathione [59], and L-homocysteine [60]. On the other hand, CBS activation by in vivo substances has not been reported. CGL activity is inhibited by acetoacetate [61], alanine [62], cysteine [63], glycine [62], serine [62], Cd^2+^ [64], Cu^2+^ [64], H_2_O_2_ [61], and O_2_ [65]. It is interesting that oxidative stress inhibits CBS activity. However, CGL is activated by L-cysteine [66] and 2-mercaptoethanol (probably other reducing agents also reactivate) [62]. Thus, reducing conditions reactivate CBS. TST activity is inhibited by Ca^2+^ [67], Zn^2+^ [67], Cu^2+^ [68], oxaloacetate [69], pyruvate [69], H_2_S [70], SO_3_^2−^ [67], SO_4_^2−^ [69], sulfide [71], sulfite [72], and H_2_O_2_ [73]. Oxidative stress also inhibits TST activity. On the other hand, TST is activated by L-cysteine [74], glutathione [75], and reduced glutathione [75,76]. Thus, reducing conditions also reactivate TST. MST activity is inhibited by alpha-ketobutyrate [77], alpha-ketoglutarate [77], pyruvate [78], cysteine [78], sulfite [6], glutathione [78], and H_2_O_2_ [79]. Oxidative stress also inhibits MST activity, while MST is activated by thioredoxin [79,80]. Thus, reducing conditions also reactivate MST. In the three enzymes, these enzymatic activities are regulated by the redox state.

## 6. Conclusions

Four cysteine-containing enzymes (CBS, CGL, MST, and TST) produce H_2_S and polysulfides via the reduction of persulfurated or polysulfurated substrates. MST is also produced via the reduction of stable persulfide (polysulfide) formed at a catalytic-site cysteine as a reaction intermediate. Sulfur oxide can also be produced from a catalytic site cysteine of MST. These products play an important role in living organisms. Furthermore, studies using KO mice of each enzyme have clarified the physiological function of these enzymes, which cannot be assessed in wild-type animals. Regulation of enzymatic activity by in vivo substances may be related to the functions of H_2_S and polysulfides.

## Figures and Tables

**Table 1:** Physiological function of H_2_S.

Function	Reference
Induction of long-term potentiation in the hippocampus as a synaptic model of memory	Abe and Kimura, 1996 [1]
Effect on smooth muscle relaxant activity	Hosoki et al., 1997 [2]
Protective action of nerve cells from oxidative stress	Kimura and Kimura, 2004 [13]
Regulation of insulin secretion	Yang et al., 2005 [14]; Kaneko et al., 2006 [15]
Oxygen sensor	Olson et al., 2006 [16]; Peng et al., 2010 [17]
Antiinfection	Zanardo et al., 2006 [18]
Protective action of myocardium and kidney from ischemia reperfusion injury	Elrod et al., 2007 [19]; Tripatara et al., 2008 [20]
Angiogenic effect	Cai et al., 2007 [21]; Papapetropoulos et al., 2009 [22]
Protection of retinal neurons from light-induced damage and apoptosis	Mikami et al., 2011b [7]
Regulation of endoplasmic reticulum stress	Krishnan et al., 2011 [23]
Bacterial resistance against antibiotics	Shatalin et al., 2011 [24]
Reduction of disulfide bonds in a ligand-binding domain of N-methyl-D-aspartic acid receptors	Kimura, 2013 [25]; 2015 [26]
Amplification of the activity of N-methyl-D-aspartic acid receptor upon activation by neurotransmitters	Kimura, 2015 [26]
Activation of H^+^-ATPase resulting in decrease of calcium influx into photoreceptor cells of the retina	Kimura, 2016 [12]

**Table 2:** Physiological function of polysulfides.

Function	Reference
Induction of calcium influx by activating a cation channel, subfamily A, and member 1 in astrocytes	Nagai et al., 2004 [27]; Kimura et al., 2013 [28]
Inhibition of tumor suppressor lipid phosphatase and tensin homolog by changing the protein to its oxidized form	Greiner et al., 2013 [29]
Upregulation of antioxidant genes, such as heme oxygenase 1 and glutamate cysteine ligase	Koike et al., 2013 [30]
Induction of long-term potentiation in the hippocampus due to activation of N-methyl-D-aspartic acid receptors	Kimura, 2015 [26]
Upregulation of antioxidant genes such as heme oxygenase 1 and glutamate cysteine ligase	Kimura, 2015 [26]
Decrease in toxic carbonyl stress in neuroblastoma cells	Koike et al., 2015 [31]
Induction of neural outgrowth and cell differentiation of neuroblastoma cells	Koike et al., 2016 [32]
